# Supplementary material for: Complex‐centric proteome profiling by SEC‐SWATH‐MS
Source: Mol Syst Biol. 2019 Jan 14;15(1):e8438. doi: 10.15252/msb.20188438 (PMC6346213; doi:10.15252/msb.20188438)
Supplement: Supplementary file 8 — Dataset EV7 [file MSB-15-e8438-s008.zip › feature_plots_string/P14625.pdf]

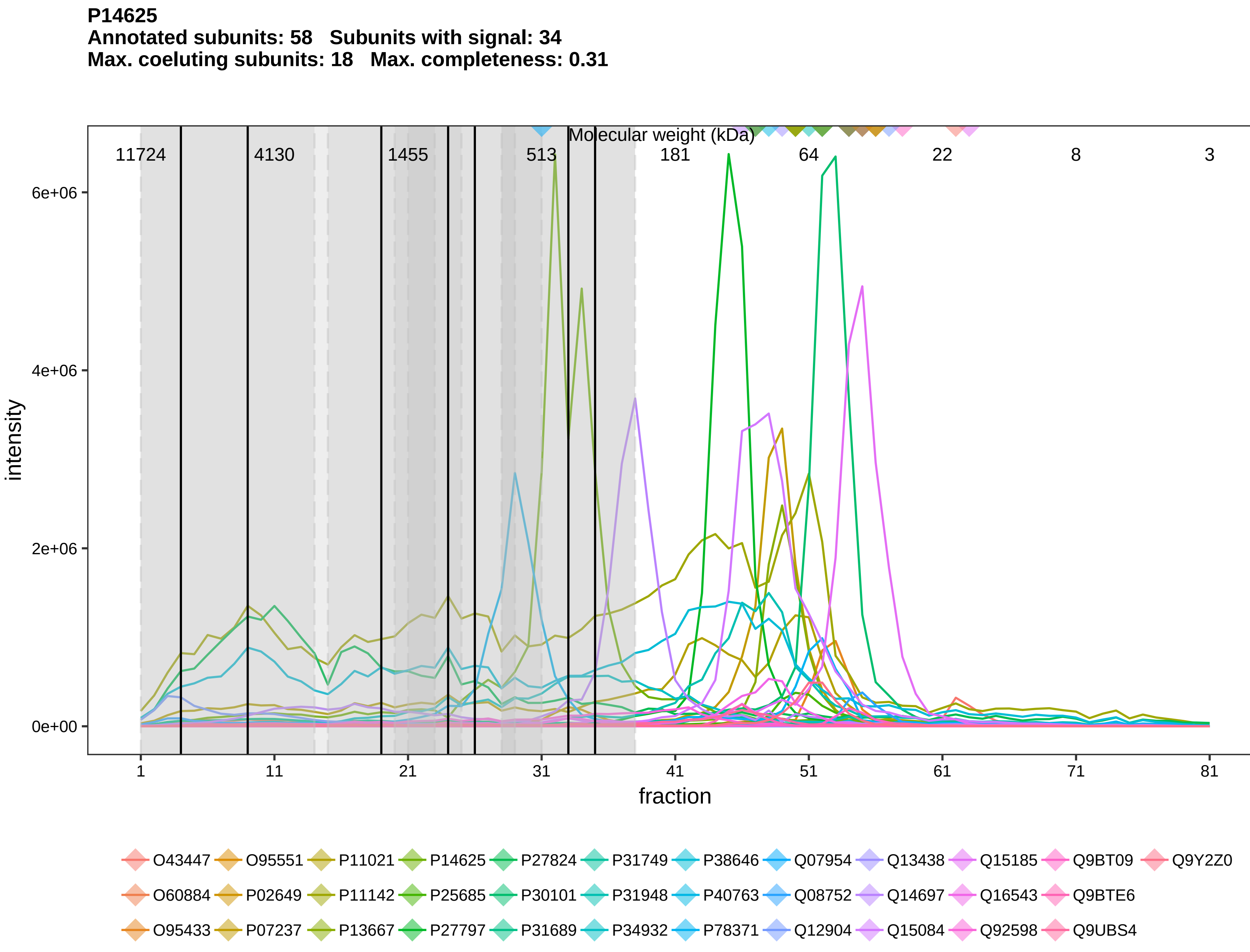

P14625
Annotated subunits: 58 Subunits with signal: 34
Max. coeluting subunits: 18 Max. completeness: 0.31
Molecular weight (kDa)
11724
4130
1455
513
181
64
22
8
3
intensity
6e+06
4e+06
2e+06
0e+00
fraction
1
11
21
31
41
51
61
71
81
O43447
O95551
P11021
P14625
P27824
P31749
P38646
Q07954
Q13438
Q15185
Q9BT09
Q9Y2Z0
O60884
P02649
P11142
P25685
P30101
P31948
P40763
Q08752
Q14697
Q16543
Q9BTE6
O95433
P07237
P13667
P27797
P31689
P34932
P78371
Q12904
Q15084
Q92598
Q9UBS4
